# Biomarker analysis of cetuximab plus oxaliplatin/leucovorin/5-fluorouracil in first-line metastatic gastric and oesophago-gastric junction cancer: results from a phase II trial of the Arbeitsgemeinschaft Internistische Onkologie (AIO)

**DOI:** 10.1186/1471-2407-11-509

**Published:** 2011-12-07

**Authors:** Birgit Luber, Joëlle Deplazes, Gisela Keller, Axel Walch, Sandra Rauser, Martin Eichmann, Rupert Langer, Heinz Höfler, Susanna Hegewisch-Becker, Gunnar Folprecht, Ewald Wöll, Thomas Decker, Esther Endlicher, Sylvie Lorenzen, Falko Fend, Christian Peschel, Florian Lordick

**Affiliations:** 1 Institut für Allgemeine Pathologie und Pathologische Anatomie, Technische Universität München, Trogerstraße 18, 81675 München, Germany; 2 Institute of Pathology, Helmholtz Zentrum München, Ingolstädter Landstraße 1, 85764 Neuherberg, Germany; 3 Onkologische Schwerpunktpraxis Eppendorf, Eppendorfer Landstraße 42, 20249 Hamburg, Germany; 4 1st Medical Department, Universitätsklinik Carl Gustav Carus, Fetscherstraße 74, 01307 Dresden, Germany; 5 Medical Department, Krankenhaus St. Vinzenz, Sanatoriumstraße 43, 6511 Zams, Austria; 6 Onkologische Schwerpunktpraxis, Wilhelm-Hauff-Straße 41, 88214 Ravensburg, Germany; 7 1st Medical Department, Klinikum der Universität, Franz-Josef-Strauß-Allee 11, 93053 Regensburg, Germany; 8 National Center of Tumor Diseases, University of Heidelberg, Im Neuenheimer Feld 460, 69120 Heidelberg, Germany; 9 Institute of Pathology, Eberhard-Karls-Universität, Liebermeisterstraße 8, 72076 Tübingen, Germany; 10 Klinikum rechts der Isar, 3rd Medical Department, Technische Universität München, Ismaninger Straße 22, 81675 München, Germany; 11 3rd Medical Department, Klinikum Braunschweig, Celler Straße 38, 38114 Braunschweig, Germany; 12 Department of Immunobiology, King's College London, London, UK

## Abstract

**Background:**

The activity of the epidermal growth factor receptor (EGFR)-directed monoclonal antibody cetuximab combined with oxaliplatin/leucovorin/5-fluorouracil (FUFOX) was assessed in first-line metastatic gastric and oesophago-gastric junction (OGJ) cancer in a prospective phase II study showing a promising objective tumour response rate of 65% and a low mutation frequency of *KRAS *(3%). The aim of the correlative tumour tissue studies was to investigate the relationship between *EGFR *gene copy numbers, activation of the EGFR pathway, expression and mutation of E-cadherin, V600E BRAF mutation and clinical outcome of patients with gastric and OGJ cancer treated with cetuximab combined with FUFOX.

**Methods:**

Patients included in this correlative study (*n *= 39) were a subset of patients from the clinical phase II study. The association between *EGFR *gene copy number, activation of the EGFR pathway, abundance and mutation of E-cadherin which plays an important role in these disorders, BRAF mutation and clinical outcome of patients was studied. *EGFR *gene copy number was assessed by FISH. Expression of the phosphorylated forms of EGFR and its downstream effectors Akt and MAPK, in addition to E-cadherin was analysed by immunohistochemistry. The frequency of mutant V600E BRAF was evaluated by allele-specific PCR and the mutation profile of the E-cadherin gene *CDH1 *was examined by DHPLC followed by direct sequence analysis. Correlations with overall survival (OS), time to progression (TTP) and overall response rate (ORR) were assessed.

**Results:**

Our study showed a significant association between increased *EGFR *gene copy number (≥ 4.0) and OS in gastric and OGJ cancer, indicating the possibility that patients may be selected for treatment on a genetic basis. Furthermore, a significant correlation was shown between activated EGFR and shorter TTP and ORR, but not between activated EGFR and OS. No V600E BRAF mutations were identified. On the other hand, an interesting trend between high E-cadherin expression levels and better OS was observed and two *CDH1 *exon 9 missense mutations (A408V and D402H) were detected.

**Conclusion:**

Our finding that increased *EGFR *gene copy numbers, activated EGFR and the E-cadherin status are potentially interesting biomarkers needs to be confirmed in larger randomized clinical trials.

**Trial registration:**

Multicentre clinical study with the European Clinical Trials Database number 2004-004024-12.

## Background

Gastric cancer was estimated to be the fourth most common cancer and second leading cause of death from cancer worldwide in 2008 [1]. During recent decades, the incidence of oesophago-gastric junction (OGJ) cancer has increased continuously [2]. Metastatic gastric and OGJ adenocarcinomas are characterised by poor prognosis and modest response to chemotherapeutic treatment [3]. Despite recent improvements in the diagnostics and therapy of these dismal diseases, new treatment options are urgently needed to achieve clinical benefits for the patients and improve their survival.

Recent advances in targeted therapy demonstrate the advantage of a combination of trastuzumab, a monoclonal antibody directed against the human epidermal growth factor receptor 2 (HER2), with chemotherapy versus chemotherapy alone in HER2-positive advanced gastric or OGJ cancer [4]. Epidermal growth factor receptor (EGFR) belongs to the same family of receptor tyrosine kinases that plays a pivotal role in the regulation of tumour cell growth and survival. Aside from HER2, EGFR may also be a promising therapeutic target in gastric cancer. Several studies have linked EGFR expression with advanced clinical stage or the presence of distant metastasis and provided evidence that EGFR may have a central role in the pathogenesis and prognosis of gastric and OGJ cancer [5,6].

Cetuximab is a monoclonal human-mouse chimeric antibody that interacts with domain III of the extracellular region of EGFR with a high specificity and inhibits ligand-induced activation [7]. Cetuximab has been approved for the treatment of advanced colorectal cancer and for use in combination with chemotherapy and with radiotherapy for the treatment of squamous cell carcinoma of the head and neck. In the first-line treatment of advanced gastric and OGJ cancer, several phase II studies assessed cetuximab in combination with different chemotherapy regimens, most of them showing promising results with objective response rates ranging from 41 to 65% [8-11].

Cetuximab combined with FUFOX showed a high response rate of 65% in first-line metastatic gastric and OGJ cancer in a prospective phase II study [10]. The expression level of EGFR on tumour cells was not correlated with therapeutic response. The aim of the present study was to investigate the relationship between *EGFR *gene copy status, activation of the EGFR pathway, and the *BRAF *mutation status with clinical outcome. Understanding the molecular basis of therapy response may require the analysis of additional markers such as the cell adhesion protein E-cadherin, which regulates epithelial-mesenchymal transition and has been associated with cetuximab response in preclinical models [12]. Therefore, the abundance of E-cadherin was determined and E-cadherin gene (*CDH1*) mutations were analysed.

## Methods

### Patient selection

The multicentre clinical study with the European Clinical Trials Database number 2004-004024-12 enrolled 52 patients from seven active centres recruited from April 2005 until March 2006 [10].

As reported earlier [10], eligibility criteria included the following: histologically confirmed metastatic or locally advanced irresectable adenocarcinoma of the stomach or OGJ; age 18 years or older; Eastern Cooperative Oncology Group (ECOG) performance status ≤ 2; ≥ 1 unidimensionally measurable lesion ≥ 1 cm in diameter detected by computed tomography (CT) scan or magnetic resonance imaging (MRI); cardiac ejection fraction within normal limits; absolute neutrophil count ≥ 2,000/μl; thrombocyte count ≥ 100,000/μl; total bilirubin ≤ 1.5 × upper limit of normal (ULN) and transaminases ≤ 2.5 × ULN; creatinine clearance > 70 ml/min; no previous malignancy and no chemotherapy except in the adjuvant or neoadjuvant setting > 6 months before study entry.

Pretherapeutic tumour material (formalin-fixed and paraffin-embedded) was obtained from 39 patients. Patients presented with advanced disease not amenable to a curative therapeutic approach. Patients gave written informed consent for translational investigations including tumour genetic analyses concerning EGFR pathway-linked genes. Data were acquired with approval from the ethics committee of the Technische Universität München.

### Treatment

As reported earlier in detail [10], cetuximab was administered at an initial dose of 400 mg/m^2 ^on day 1 over 120 mins, followed by weekly doses of 250 mg/m^2 ^over 60 mins. Oxaliplatin 50 mg/m^2 ^was given i.v. over 120 min followed by folinic acid 200 mg/m^2 ^i.v. over 120 min and 5-fluorouracil 2,000 mg/m^2 ^i.v. over 24 h on days 1, 8, 15, and 22, every 5 weeks (1 cycle). Treatment continued until best response, or until there was evidence of disease progression, unacceptable toxicity, death, or withdrawal of patient consent. Toxicity was graded according to National Cancer Institute Common Toxicity Criteria (NCI-CTC, version 3.0).

### Response

Clinical response was determined by computer tomography according to the Response Evaluation Criteria in Solid Tumours (RECIST) as reported earlier [10].

### Fluorescence in situ hybridisation

The *EGFR *gene copy status and the ploidy status of chromosome 7 were evaluated in tumour specimen of 36 patients by image-based three-dimensional fluorescence in situ hybridisation (FISH). FISH analysis was performed on 16-μm paraffin sections as previously described [13,14]. The commercially available LSI EGFR SpectrumOrange/CEP 7 SpectrumGreen Dual Color Probe (Abbott, Wiesbaden, Germany) was applied according to the manufacturer's instructions. Signals were evaluated by optical sectioning and three-dimensional imaging as previously described [15]. Tumours were classified into the following categories with respect to their mean *EGFR *gene copy numbers: (1) < 2.5 (normal), (2) ≥ 2.5- < 4.0 (low level copy number gain), (3) ≥ 4.0- < 6.0 (high level copy number gain), (4) ≥ 6.0 (amplification). Tumours were also classified into the following categories with respect to their mean chromosome 7 centromeric signals (*CEP7*) copy numbers: (1) < 2.5, (2) ≥ 2.5- < 4.0, (3) ≥ 4.0- < 6.0 and (4) ≥ 6.0.

### Antibodies

The following antibodies were used for immunohistochemistry: anti-phosphorylated EGFR (pEGFR) rabbit polyclonal antibody reacting with EGFR only when phosphorylated at tyrosine residue 1086 (#36-9700, obtained from Zymed Laboratories, distributed by Invitrogen, Karlsruhe, Germany), anti-phosphorylated Akt (pAkt) mouse monoclonal antibody recognising Akt phosphorylated at Ser473 (#4051, Cell Signaling Technology, distributed by New England Biolabs, Frankfurt, Germany), anti-phosphorylated p44/42 MAPK (pMAPK) rabbit polyclonal antibody detecting p44/42 MAPK only when phosphorylated at Thr202/Tyr204 (#9101, Cell Signaling Technology) and anti-E-cadherin mouse monoclonal antibody HECD-1 (# ALX-804-201, Alexis Biochemicals, distributed by Axxora Deutschland GmbH, Lörrach, Germany).

### Immunohistochemical analysis

EGFR expression was determined using a standardised immunohistochemistry assay (EGFR pharmDx Dako, Glostrup, Denmark) as reported earlier [10]. A manual staining protocol was used for pEGFR, pAkt, pMAPK and E-cadherin as described in Additional file 1.

### Reactivity score and interpretation of the immunohistochemical staining

Evaluation of the immunhistochemical biomarker staining was performed by at least two investigators (A.W. and B.L.) who were unaware of clinical features and survival. The reactivity score and the interpretation of the staining are described in Additional file 1.

### DNA extraction

The protocol for DNA extraction is available in Additional file 1.

### BRAF mutation analysis

The BRAF hotspot mutation V600E was analysed using allele-specific PCR following an established protocol [16]. Positive and negative controls which had been determined by direct sequencing were included in this analysis.

### *CDH1 *mutation analysis

Structural alterations of exons 2-16 of the *CDH1 *gene were analysed in the tumours of 22 patients by denaturing high performance liquid chromatography (DHPLC) as described previously [17]. Upon detection of aberrant DHPLC patterns, DHPLC analysis was followed by direct sequencing of the PCR products. Additional information is available in Additional file 1.

### Statistical analysis

As reported earlier [10], the primary study endpoint was the proportion of patients who responded to cetuximab-FUFOX. The study was designed as a two-stage trial assuming a response rate of ≤ 30% as not being of further interest (null hypothesis) and a response rate of ≥ 50% as interesting (alternative hypothesis). The best observed response was taken as a basis for determination of the primary endpoint and confirmed responses were also reported. Treatment with cetuximab plus FUFOX induced objective tumour responses in 65% of treated patients which was clearly above the threshold for accepting the alternative hypothesis.

The calculation of TTP, OS, duration of treatment and follow-up is explained in Additional file 1. Kaplan-Meier survival time analysis was used to correlate the investigated markers with TTP and OS. Differences in survival between subgroups were compared by log-rank test. Cox regression analysis was performed correlating the biomarkers with TTP and OS. Hazard ratios (HR) calculated by the Cox proportional hazards model were reported with 95% CIs. The association between the biomarkers and ORR or clinical benefit rate (CBR), respectively, was studied using Fisher's exact and *χ*^2 ^tests when appropriate. Correlation analysis between the investigated markers was performed using the Pearson test and Spearman's rho test. A two-sided p value less than 0.05 was considered to be statistically significant. The statistical analysis was carried out with SPSS V17.0 software (SPSS Inc., Chicago, IL, USA).

## Results

### Patient and tumour characteristics and clinical outcome

Patients with advanced gastric and OGJ cancer were a subset of 52 patients who were enrolled in the clinical trial between April 2005 and March 2006. Pretherapeutic tumour material was available from 39 patients. The location of the primary tumour was the OGJ in 22 (56%) patients and other parts of the stomach in the remaining 17 (44%) patients (Table 1). As reported previously, all patients presented with metastatic disease, with lymph nodes, liver, peritoneum and lung as the predominant metastatic sites [10]. The Laurén classification of the tumours was intestinal in 22 (56%) cases and non-intestinal (diffuse or mixed type) in 17 (44%) cases.

**Table 1:** Patient and tumour characteristics

Characteristic	No. of Patients (n = 39)	%
**Age (years)**		

Median	63	

Range	38-80	

**Gender**		

Male	30	77

Female	9	23

**ECOG performance status**		

0	16	41

1	17	44

2	6	15

**Disease status**		

Locally advanced	0	0

Metastatic	39	100

**Site of the primary tumour**		

Oesophago-gastric junction	22	56

Stomach	17	44

**Histological classification (Laurén)**		

Intestinal	22	56

Non-intestinal (diffuse or mixed)	17	44

Abbreviation: *ECOG *Eastern Cooperative Oncology Group

The median OS was 293 days (95% CI, 202 days to 384 days) with a 1-year survival rate of 38%. The median TTP was 230 days (95% CI, 144 days to 316 days) with a probability of remaining progression-free at 1 year of 10%. The overall response rate (ORR; complete response + partial response) of this patient subset was 62% (95% CI: 46-76%), including 3 complete and 20 partial responses. Clinical benefit rate (CBR; complete response + partial response + stable disease) was 81% (95% CI: 66-90%). Median duration of treatment was 106 days; median follow-up was 379 days.

### Relationship between *EGFR *gene copy numbers and therapy response

**Figure 1:**
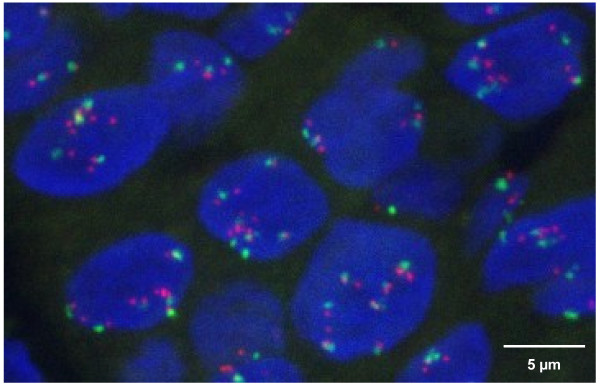
**Detection of *EGFR *gene amplification and survival impact of *EGFR *copy number gain**. (**a**) *EGFR *gene amplification (8.2 signals per nucleus) was observed in one patient with OGJ cancer by image-based three-dimensional dual colour FISH analysis for *EGFR *(red) and chromosome 7 (green). (**b**) Overall survival in metastatic gastric or OGJ cancer patients treated with cetuximab plus FUFOX was analysed with the Kaplan-Meier method after stratification of patients according to *EGFR *gene copy numbers (cut-off 4.0). The log-rank test statistical analysis indicates a statistically significant relationship (*P *= 0.011).

*EGFR *gene copy numbers and *CEP7 *copy numbers were determined by three-dimensional image-based FISH in gastric and OGJ tumours from 36 patients. The range of *EGFR *gene copy numbers was 2.0-8.2 (signals per nucleus). Tumours were classified into four categories with respect to their mean *EGFR *gene copy numbers. The majority of tumours revealed *EGFR *low level copy number gain (56%), while high level copy number gain was less frequent (19%), and amplification was rare (3%) (Figure 1a, Table 2). The *CEP7 *copy numbers (signals per nucleus) were in the range of 2.0-6.0. In most of the tumours, low copy number changes for *CEP7 *(≥ 2.5- < 4.0) were observed (61%). The *EGFR/CEP7 *ratio was within the range 0.7-1.5. The majority of patients were in the group < 1.1 (61%).

**Table 2:** *EGFR *gene copy analysis by three-dimensional FISH

No. of Patients (%), (n = 36)				
**Mean copy number**	**< 2.5**	**≥ 2.5- < 4.0**	**≥ 4.0- < 6.0**	**≥ 6.0**
	**(normal)**	**(low level)**	**(high level)**	**(amplification)**

*EGFR*	8 (22%)	20 (56%)	7 (19%)	1 (3%)

*CEP7*	11 (31%)	22 (61%)	2 (6%)	1 (3%)

**Ratio**	< 1.1	≥ 1.1-1.5		

*EGFR/CEP7*	22 (61%)	14 (39%)		

Abbreviation: *FISH *fluorescence *in situ *hybridisation

*EGFR *gene copy numbers were correlated with *CEP7 *signals (Pearson test *P *< 0.001; Spearman's rho test *P *< 0.001) and *EGFR/CEP7 *ratio (Pearson test *P *= 0.013; Spearman's rho test *P *= 0.002) (Additional file 1: Supplementary Tables S1 and S2).

Increased *EGFR *gene copy number (≥ 4.0) was significantly associated with better OS (log-rank *P *= 0.011; HR 0.2, 95% CI: 0-0.8; *P *= 0.022) (Figure 1b, Additional file 1: Supplementary Table S3). Increased *CEP7 *gene copy number (≥ 3.0 signals, aneusomy) was also related to OS (log-rank *P *= 0.025; HR 0.4, 95% CI: 0.2-0.9; *P *= 0.030). In contrast, it was not possible to define a cut-off point that demonstrated a relationship between *EGFR/CEP7 *ratio and OS.

Copy numbers of the *EGFR *gene and *CEP7 per se *were not associated with TTP (Additional file 1: Supplementary Table S4). In contrast, the *EGFR/CEP7 *ratio was significantly related to TTP using the Kaplan-Meier method (log-rank *P *= 0.045), while the significance level was not reached in univariate Cox regression analysis (HR 0.4, 95% CI: 0.1-1.0; *P *= 0.056). No association was found between *EGFR *and *CEP7 *gene copy number and *EGFR/CEP7 *ratio with ORR or CBR (Additional file 1: Supplementary Table S5).

### Correlation between activation of the EGFR signalling pathway and clinical outcome

Expression levels of the phosphorylated forms of EGFR (pEGFR, Y1086), Akt kinase (pAkt, Ser473) and mitogen-activated protein kinase (pMAPK, Thr202/Tyr204) were determined by means of immunohistochemistry (Additional file 1: Supplementary Table S8). The examination of the EGFR expression level was performed and previously reported [10]. The results obtained by this analysis were included in the present study to search for correlations with the presently analysed biomarkers and clinical outcome. In total, pretherapeutic biopsies of 28 patients were available for the immunohistochemical study. Staining of pEGFR was interpretable in 26 cases (93%), pAkt in 28 cases (100%), pMAPK in 25 cases (89%) and E-cadherin in 25 cases (89%).

Correlation analysis revealed a significant association between EGFR and pEGFR expression (*P *= 0.040, Pearson test) (Additional file 1: Supplementary Table S1). pEGFR staining was not linked to pAkt or pMAPK staining.

No statistically significant association between EGFR, pEGFR, pAkt or pMAPK expression levels and OS was found (Additional file 1: Supplementary Table S3). A significant correlation was shown between pEGFR and shorter TTP (log-rank *P *= 0.018; HR 4.0, 95% CI: 1.2-13.9, *P *= 0.027), but not between EGFR, pAkt or pMAPK and TTP (Additional file 1: Supplementary Table S4). The pEGFR expression level was associated with ORR but not with CBR (Table 3, Additional file 1: Supplementary Table S5): ORR was 31% in patients with pEGFR-positive tumours and 92% in patients with pEGFR-negative tumours (*P *= 0.004), clinical benefit (CR, PR and SD) was achieved in 92% of patients who presented with pEGFR-negative tumours and 62% of patients with pEGFR-positive carcinomas (*P *= 0.160). Median TTP was 121 days in patients with pEGFR-positive tumours and 291 days in patients with pEGFR-negative tumours (*P *= 0.018). Median OS of patients suffering from pEGFR positive tumours was 285 days and 490 days in patients with pEGFR-negative tumours (*P *= 0.619).

**Table 3:** Clinical outcome according to pEGFR immunohistochemistry

	pEGFR detectable	pEGFR non detectable	*P *value
Overall response rate (CR and PR)	31%	92%	0.004*

Clinical benefit rate (CR, PR and SD)	62%	92%	0.160*

Median time to progression	121 days	291 days	0.018**

Median overall survival	285 days	490 days	0.619**

*Fisher's exact test, two-sided

**log-rank *P*

Oncogenic activation of EGFR downstream effectors in the RAS-RAF-MAPK signalling pathway can negatively influence cetuximab therapy. As reported earlier, *KRAS *mutations were examined by direct sequencing of exon 2 [10]. One single *KRAS *mutation at nucleotide position 35 (G > A, Patient 36) that changes codon 12 from glycine to aspartic acid (G12D) was detected in 1 of 32 patients (3%, Table 4). The clinical benefit of the therapy was stable disease, which means that the patient was, by definition, a non-responder (Additional file 1: Supplementary Table S10). This *KRAS *mutation was previously described in gastric cancer [18]. In addition, the hotspot mutation V600E of the BRAF protein was analysed in the present study using allele-specific PCR. No V600E BRAF mutation was found in 32 patients (Table 4). Together, the mutation frequency of key signalling molecules of the RAS-RAF-MAPK pathway in this patient cohort was very low.

**Table 4:** Summary of mutation analysis

Gene	No. of patients	No. of mutations	%
*KRAS*	32	1	3

*BRAF*	32	0	0

*CDH1*	22	2	9

### Influence of the E-cadherin expression level or mutation status on treatment outcome

To determine the contribution of expression or mutation of E-cadherin to clinical outcome, immunohistochemical detection of E-cadherin protein expression and mutation profiling of exons 2-16 of the E-cadherin gene *CDH1 *were performed.

**Figure 2:**
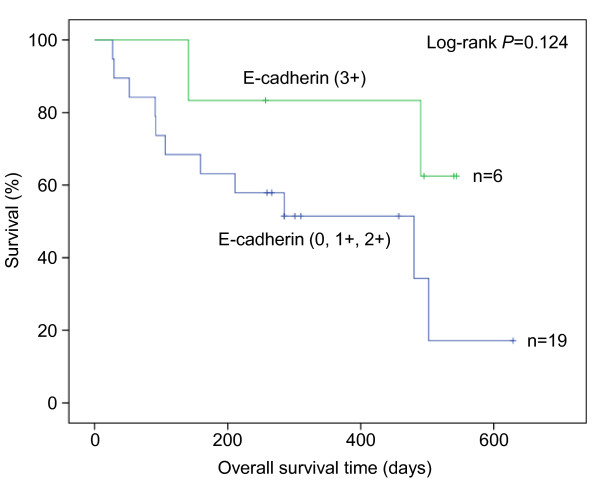
**Survival impact of E-cadherin expression and sequence analysis of *CDH1 *exon 9 missense mutations**. (**a**) Overall survival in metastatic gastric or OGJ cancer patients treated with cetuximab plus FUFOX was analysed by the Kaplan-Meier method after stratification of patients according to their E-cadherin expression level (score 0, 1+, 2+ versus 3+). The log-rank test statistical analysis indicates a trend between high E-cadherin expression levels and increased OS (*P *= 0.124). (**b**) In the tumours of two patients, *CDH1 *exon 9 missense mutations were detectable and caused amino acid changes D402H or A408V.

An interesting trend between high E-cadherin expression levels and increased OS was observed (log-rank *P *= 0.124; HR 0.3, 95% CI: 0.1-1.5, *P *= 0.141) (Additional file 1: Supplementary Table S3, Figure 2a). No correlations were found between E-cadherin expression and TTP, ORR or CBR.

*CDH1 *missense mutations in exon 9 were detected in 2 of 22 tumours (9%), and several polymorphisms were found in 4 of 22 tumours (18%) (Additional file 1: Supplementary Table S9). In total, 15 of 22 analysed tumours were of the non-intestinal type (diffuse or mixed type). The *CDH1 *missense mutations occurred in 2 out of 15 tumours (13%) of these histological subtypes and were diffuse-type signet cell ring gastric carcinomas. Both mutations were confirmed by sequencing and not detected in the adjacent normal tissues from the same patients (Figure 2b).

The first tumour showed a *CDH1 *missense mutation in exon 9 (Patient 27) at nucleotide position c1204 that changes codon 402 from aspartic acid to histidine, thereby affecting the putative calcium-binding motif DAD (DAD > DAH). The second tumour harboured a novel *CDH1 *missense mutation in exon 9 (Patient 11) at nucleotide position c1223 that changes codon 408 from alanine to valine.

Both tumours (with *CDH1 *missense mutation in exon 9) occurred in two female patients with peritoneal carcinogenesis. Due to early disease progression, their survival times after first infusion with cetuximab were very short (only 27 days for Patient 11 and 29 days for Patient 27). Consequently, the response rate of Patient 11 was not evaluable (Additional file 1: Supplementary Table S10). Patient 27 was classified as a non-responder.

## Discussion

Here, we report the results of a correlative research program from a multicentre phase II study of cetuximab plus weekly oxaliplatin, 5-fluorouracil and folinic acid in first-line advanced gastric and OGJ cancer.

### Predictive value of *EGFR *gene copy number gain

In gastric and OGJ cancer, the optimal cut-off points to study an association between *EGFR *gene copy number and clinical outcome during cetuximab treatment have not been established. This fact explains the requirement of a statistical search for the best cut-offs by correlating the *EGFR *status with overall patient survival using the log rank test. We found that the optimal cut-off value that separated patients with favourable and unfavourable prognosis was a mean number of 4.0 *EGFR *signals per nucleus.

We provide evidence that increased *EGFR *and *CEP7 *copy numbers were associated with better OS of patients with metastatic gastric or OGJ cancer treated with cetuximab and chemotherapy, but it was not possible to define a cut-off point that showed a relationship between *EGFR/CEP7 *ratio and OS. Although tumour growth in responsive patients may be closely linked to an increase in *EGFR/CEP7 *ratio, the literature in other tumour entities, especially in metastatic colorectal cancer demonstrates the experimental difficulties in finding a valid cut-off [19].

Several studies have shown a relationship between specific molecular alterations of the *EGFR *gene, including somatic mutations and copy number variations, and the clinical activity of anti-EGFR drugs. Somatic *EGFR *mutations have been reported to be the genetic events underlying responsiveness of non-small cell lung cancer to the small molecular EGFR-inhibitor gefitinib [20]. Increased copy number of the *EGFR *gene is associated with response to cetuximab in colorectal cancer patients [21]. However, in gastric cancer, *EGFR *gene amplification only occurs in a subset of patients in the range of 2-9% [22-24]. In accordance with these studies, we found a low frequency of *EGFR *gene amplification (3%). Interestingly, we detected a high frequency of *EGFR *low copy gain (56%), which can only be reliably detected by image-based three-dimensional FISH in thick (16 μm) sections and not by standard FISH in thin (4 μm) sections, as was used in previous studies [15]. Notably, we found that diploid cases in our study were predominantly of the intestinal type, and in contrast to the situation in colorectal cancer, the diploid genotype was not predictive of therapy response. In accordance with the low prevalence of genetic amplification of the *EGFR *gene in this type of cancer, none of the 38 patients exhibited increased *EGFR *gene copy number in a recent phase II trial of cetuximab in combination with mFOLFOX6 in gastric and OGJ cancer [25].

### Influence of the EGFR signalling cascade on patient outcome

Because cetuximab binds to the extracellular domain of EGFR, the obvious assumption was that the presence of EGFR on gastric cancer cell membranes would be predictive for response to cetuximab. However, the expression level of EGFR on tumour cells *per se*, as determined by immunohistochemistry, was not predictive of therapeutic response in gastric and OGJ cancer in various phase II trials of cetuximab in combination with chemotherapeutic agents [8,10,25]. This observation is reminiscent of the situation found in colorectal cancer, where immunohistochemical measurement of EGFR expression may not be an accurate predictive factor for response to cetuximab therapy [26]. Notably, advanced gastric cancer patients with EGFR expression in their tumours together with low serum levels of the ligands EGF and transforming growth factor-α showed better response in one study [25]. Recent data on EGFR expression and clinical outcome of patients treated with cetuximab plus irinotecan/leucovorin/5-fluorouracil showed that tumour response was more commonly found in advanced OGJ cancer patients whose tumours expressed EGFR, although EGFR expression was not associated with improved progression-free survival or overall survival [11]. Taken together, the reports on EGFR expression and clinical outcome of gastric and OGJ cancer patients treated with cetuximab plus chemotherapy are not consistent.

Activation of EGFR triggers a signalling cascade that comprises essentially two downstream pathways: the RAS-RAF-MAPK axis is mainly involved in cell proliferation, and the PI3K-Akt axis primarily controls cell survival [27]. The results of the present study suggest that the pEGFR expression level was associated with clinical outcome. We found that in patients with pEGFR-positive tumours, TTP was significantly shorter compared with those patients without detectable pEGFR in their tumours and that the pEGFR expression level was associated with the ORR. However, pEGFR expression was not significantly correlated with OS. Differences in OS may have been blurred by subsequent therapies not containing EGFR-antagonists. In an attempt to better understand the mechanisms of resistance to EGFR inhibitors, cell lines resistant to EGFR inhibitors were reported to reveal elevated levels of pEGFR, pAkt and signal transducer and activator of transcription 3, which were associated with reduced apoptotic capacity [28]. In our study, pEGFR expression was not correlated with pAkt or pMAPK expression, suggesting that activation of the PI3K-Akt cascade and the MAPK pathway occurred independently of EGFR.

Colorectal cancers lacking oncogenic alterations in genes encoding EGFR downstream effectors such as KRAS, BRAF, PIK3CA, and PTEN have the highest probability of response to anti-EGFR therapies [27,29]. In contrast to colorectal cancer, the prevalence of *KRAS *mutations in gastric cancer is low [22] and consequently, a relationship between *KRAS *mutations and therapy response is difficult to establish. The frequency of *KRAS *mutations was between 0 and 9% in several phase II trials of cetuximab in combination with chemotherapeutic agents in advanced gastric or OGJ adenocarcinoma [9-11,25]. We did not detect any V600E BRAF mutations in our study while the frequency of *BRAF *mutations was 12% in the aforementioned study of cetuximab plus chemotherapy in advanced OGJ cancer patients [11]. Together, in contrast to the situation in colorectal cancer, oncogenic alterations in genes encoding signalling molecules of the RAS-RAF-MAPK pathway are rare in gastric and OGJ cancer.

### Predictive role of E-cadherin expression and somatic *CDH1 *gene mutations

Activation of EGFR is negatively influenced by E-cadherin-mediated cell adhesion [30]. Therefore, we hypothesised that E-cadherin affects the response to cetuximab plus chemotherapy of gastric and OGJ cancer patients. We found that patients with high expression of E-cadherin in their tumours have an overall survival advantage compared to patients with moderate or low expression. However, this result did not reach statistical significance, presumably due to the low number of investigated patients. In several studies, cancer cell lines with high E-cadherin expression levels were found to be more susceptible to cetuximab or other anti-EGFR agents than cell lines with low E-cadherin levels [12,31].

Because E-cadherin mutations are frequently detected in somatic and germline diffuse-type gastric cancer [17,32], we also decided to assess whether the *CDH1 *mutation status is crucial to predict clinical outcome. We have previously shown that certain *CDH1 *mutations have a negative influence on survival of gastric carcinoma patients [33] and that *CDH1 *mutations are associated with enhanced EGFR activation [34,35].

We detected the *CDH1 *mutations D402H and A408V in diffuse-type signet cell ring gastric carcinomas, confirming previous observations that *CDH1 *mutations are associated with diffuse- or mixed-type gastric cancer but not with intestinal gastric carcinoma [32]. The first of these mutations has been described previously at the amino acid level but at a different nucleotide position [36]. The second mutation has not been previously described. The total frequency of *CDH1 *mutations in exons 2-16 that were observed in our study was 9% for all investigated tumours and 13% when only diffuse and mixed-type gastric carcinomas were taken into account. *CDH1 *mutation frequencies for advanced diffuse-type gastric carcinomas reported from other studies are 28% [37], 50% [32] and 70% [38]. Due to the low mutation frequency and early disease progression observed here, a correlation between the occurrence of *CDH1 *mutations and clinical outcome cannot be calculated.

The prognostic significance of E-cadherin expression in gastric carcinoma is under controversial discussion [39,40]. To our knowledge, this study is the first to assess the abundance and genetic mutation of E-cadherin as biomarkers of response to EGFR-targeted therapy. Larger studies are required to assess the contribution of E-cadherin expression and *CDH1 *mutations to clinical outcome of gastric and OGJ cancer patients treated with anti-EGFR therapy.

## Conclusions

Specific alterations of the *EGFR *gene, including copy number variations, and oncogenic activation of EGFR downstream effectors such as KRAS and BRAF had been previously reported as the genetic events underlying the response to cetuximab plus chemotherapy in colorectal cancer. Our study showed an association between *EGFR *gene copy number and survival in gastric and OGJ cancer, suggesting that patients may be selected for treatment on a genetic basis. A significant correlation was shown between pEGFR and shorter TTP, but not between pEGFR and OS. On the other hand, an interesting trend between high E-cadherin expression levels and better OS was observed and two *CDH1 *missense mutations in exon 9 (A408V and D402H) were identified. However, due to the low mutation frequency and early disease progression, the relationship between the occurrence of *CDH1 *mutations and clinical outcome could not be assessed.

Taken together, we consider the *EGFR *gene copy status, activated EGFR and E-cadherin as promising candidate biomarkers. Due to the small number of patients studied, this trial has to be considered as a hypothesis-generating study and the results need to be confirmed in independent randomized clinical trials.

The efficacy of cetuximab plus capecitabine and cisplatin in advanced gastric and OGJ cancer is currently being investigated in the multinational "Erbitux in combination with Xeloda and cisplatin in advanced esophago-gastric cancer" (EXPAND) phase III trial (NCT00678535).

## Abbreviations

CBR: Clinical benefit rate; CC: Correlation coefficient; CI: Confidence interval; CT: Computed tomography; DHPLC: Denaturing high performance liquid chromatography; ECOG: Eastern Cooperative Oncology Group; EGFR: Epidermal growth factor receptor; FUFOX: Oxaliplatin/leucovorin/5-fluorouracil; HER2: Human epidermal growth factor receptor 2; HR: Hazard ratio; UICC: International Union Against Cancer; MAPK: Mitogen-activated protein kinase; MRI: Magnetic resonance imaging; NCBI: National Center for Biotechnology Information; OGJ: Oesophago-gastric junction; ORR: Overall response rate; OS: Overall survival; pAKT: Phosphorylated Akt/protein kinase B; pEGFR: Phosphorylated epidermal growth factor receptor; PI3K: Phosphatidylinositol 3-kinase; pMAPK: Phosphorylated mitogen-activated protein kinase; PCR: Polymerase chain reaction; RR: Relative risk; TTP: Time to progression; TNM: Tumour node metastasis; ULN: Upper limit of normal

## Competing interests

Florian Lordick has received research support and lecture honoraria from Merck KGaA and from Sanofi-Aventis GmbH Germany. Gunnar Folprecht has received honoraria for advisory boards from Merck KGaA, Sanofi-Aventis, Bristol-Myers-Squibb and Roche, lecture honoraria from Merck KGaA, Novartis and Amgen and a study grant from Merck KGaA.

## Authors' contributions

FL was the principal investigator of the clinical phase II trial. BL and FL designed the molecular analysis. JD carried out the mutation screening. GK supervised the mutation analysis and participated in the sequence alignment. AW, RL and FF were involved in the immunohistochemical analysis, AW and RL evaluated the stainings. AW, SR and ME carried out and interpreted the FISH analysis. SHB, GF, EW, TD, EE, SL, FF, CP and HH were involved in the phase II study, in the collection of tissue samples and in the acquisition and interpretation of clinical data. BL performed the statistical analysis. BL and FL drafted the manuscript. All authors read and improved the final manuscript.

## Pre-publication history

The pre-publication history for this paper can be accessed here:

http://www.biomedcentral.com/1471-2407/11/509/prepub

## Supplementary Material

Additional file 1 **The file Supplementary-BMC Cancer in the PDF format contains supplementary results, methods and tables**. Click here for file

## References

[B1] Ferlay J Shin HR Bray F Forman D Mathers C Parkin DM Estimates of worldwide burden of cancer in 2008: GLOBOCAN 2008 Int J Cancer 2010 127 12 2893 2917 10.1002/ijc.25516 21351269

[B2] Demeester SR Epidemiology and biology of esophageal cancer Gastrointest Cancer Res 2009 3 2 Suppl S2 S5 PMC2684731 19461918

[B3] Shah MA Kelsen DP Gastric cancer: a primer on the epidemiology and biology of the disease and an overview of the medical management of advanced disease J Natl Compr Canc Netw 2010 8 4 437 447 10.6004/jnccn.2010.0033 20410336

[B4] Bang YJ Van Cutsem E Feyereislova A Chung HC Shen L Sawaki A Lordick F Ohtsu A Omuro Y Satoh T Trastuzumab in combination with chemotherapy versus chemotherapy alone for treatment of HER2-positive advanced gastric or gastro-oesophageal junction cancer (ToGA): a phase 3, open-label, randomised controlled trial Lancet 2010 376 9742 687 697 10.1016/S0140-6736(10)61121-X 20728210

[B5] Gamboa-Dominguez A Dominguez-Fonseca C Quintanilla-Martinez L Reyes-Gutierrez E Green D Angeles-Angeles A Busch R Hermannstadter C Nahrig J Becker KF Epidermal growth factor receptor expression correlates with poor survival in gastric adenocarcinoma from Mexican patients: a multivariate analysis using a standardized immunohistochemical detection system Mod Pathol 2004 17 5 579 587 10.1038/modpathol.3800085 15073595

[B6] Nicholson RI Gee JM Harper ME EGFR and cancer prognosis Eur J Cancer 2001 37 Suppl 4 S9 S15 10.1016/s0959-8049(01)00231-3 11597399

[B7] Li S Schmitz KR Jeffrey PD Wiltzius JJ Kussie P Ferguson KM Structural basis for inhibition of the epidermal growth factor receptor by cetuximab Cancer Cell 2005 7 4 301 311 10.1016/j.ccr.2005.03.003 15837620

[B8] Pinto C Di Fabio F Siena S Cascinu S Rojas Llimpe FL Ceccarelli C Mutri V Giannetta L Giaquinta S Funaioli C Phase II study of cetuximab in combination with FOLFIRI in patients with untreated advanced gastric or gastroesophageal junction adenocarcinoma (FOLCETUX study) Ann Oncol 2007 18 3 510 517 10.1093/annonc/mdl459 17164226

[B9] Pinto C Di Fabio F Barone C Siena S Falcone A Cascinu S Rojas Llimpe FL Stella G Schinzari G Artale S Phase II study of cetuximab in combination with cisplatin and docetaxel in patients with untreated advanced gastric or gastro-oesophageal junction adenocarcinoma (DOCETUX study) Br J Cancer 2009 101 8 1261 1268 10.1038/sj.bjc.6605319 PMC2768436 19773760

[B10] Lordick F Luber B Lorenzen S Hegewisch-Becker S Folprecht G Woll E Decker T Endlicher E Rothling N Schuster T Cetuximab plus oxaliplatin/leucovorin/5-fluorouracil in first-line metastatic gastric cancer: a phase II study of the Arbeitsgemeinschaft Internistische Onkologie (AIO) Br J Cancer 2010 102 3 500 505 10.1038/sj.bjc.6605521 PMC2822949 20068568

[B11] Moehler M Mueller A Trarbach T Lordick F Seufferlein T Kubicka S Geissler M Schwarz S Galle PR Kanzler S Cetuximab with irinotecan, folinic acid and 5-fluorouracil as first-line treatment in advanced gastroesophageal cancer: a prospective multi-center biomarker-oriented phase II study Ann Oncol 2010 22 6 1358 1366 10.1093/annonc/mdq591 21119032

[B12] Black PC Brown GA Inamoto T Shrader M Arora A Siefker-Radtke AO Adam L Theodorescu D Wu X Munsell MF Sensitivity to epidermal growth factor receptor inhibitor requires E-cadherin expression in urothelial carcinoma cells Clin Cancer Res 2008 14 5 1478 1486 10.1158/1078-0432.CCR-07-1593 18316572

[B13] Walch A Bink K Hutzler P Bowering K Letsiou I Zitzelsberger H Braselmann H Stein H Hofler H Werner M Sequential multilocus fluorescence in situ hybridization can detect complex patterns of increased gene dosage at the single cell level in tissue sections Lab Invest 2001 81 10 1457 1459 10.1038/labinvest.3780359 11598158

[B14] Walch A Specht K Bink K Zitzelsberger H Braselmann H Bauer M Aubele M Stein H Siewert JR Hofler H Her-2/neu gene amplification, elevated mRNA expression, and protein overexpression in the metaplasia-dysplasia-adenocarcinoma sequence of Barrett's esophagus Lab Invest 2001 81 6 791 801 10.1038/labinvest.3780289 11406641

[B15] Rauser S Weis R Braselmann H Feith M Stein HJ Langer R Hutzler P Hausmann M Lassmann S Siewert JR Significance of HER2 low-level copy gain in Barrett's cancer: implications for fluorescence in situ hybridization testing in tissues Clin Cancer Res 2007 13 17 5115 5123 10.1158/1078-0432.CCR-07-0465 17785566

[B16] Loughrey MB Waring PM Tan A Trivett M Kovalenko S Beshay V Young MA McArthur G Boussioutas A Dobrovic A Incorporation of somatic BRAF mutation testing into an algorithm for the investigation of hereditary non-polyposis colorectal cancer Fam Cancer 2007 6 3 301 310 10.1007/s10689-007-9124-1 17453358

[B17] Keller G Vogelsang H Becker I Plaschke S Ott K Suriano G Mateus AR Seruca R Biedermann K Huntsman D Germline mutations of the E-cadherin(CDH1) and TP53 genes, rather than of RUNX3 and HPP1, contribute to genetic predisposition in German gastric cancer patients J Med Genet 2004 41 6 e89 10.1136/jmg.2003.015594 PMC1735803 15173255

[B18] Lee SH Lee JW Soung YH Kim HS Park WS Kim SY Lee JH Park JY Cho YG Kim CJ BRAF and KRAS mutations in stomach cancer Oncogene 2003 22 44 6942 6945 10.1038/sj.onc.1206749 14534542

[B19] Moroni M Sartore-Bianchi A Veronese S Siena S EGFR FISH in colorectal cancer: what is the current reality? Lancet Oncol 2008 9 5 402 403 10.1016/S1470-2045(08)70109-8 18452847

[B20] Lynch TJ Bell DW Sordella R Gurubhagavatula S Okimoto RA Brannigan BW Harris PL Haserlat SM Supko JG Haluska FG Activating mutations in the epidermal growth factor receptor underlying responsiveness of non-small-cell lung cancer to gefitinib N Engl J Med 2004 350 21 2129 2139 10.1056/NEJMoa040938 15118073

[B21] Personeni N Fieuws S Piessevaux H De Hertogh G De Schutter J Biesmans B De Roock W Capoen A Debiec-Rychter M Van Laethem JL Clinical usefulness of EGFR gene copy number as a predictive marker in colorectal cancer patients treated with cetuximab: a fluorescent in situ hybridization study Clin Cancer Res 2008 14 18 5869 5876 10.1158/1078-0432.CCR-08-0449 18794099

[B22] Kim IJ Park JH Kang HC Shin Y Park HW Park HR Ku JL Lim SB Park JG Mutational analysis of BRAF and K-ras in gastric cancers: absence of BRAF mutations in gastric cancers Hum Genet 2003 114 1 118 120 10.1007/s00439-003-1027-0 14513361

[B23] Moutinho C Mateus AR Milanezi F Carneiro F Seruca R Suriano G Epidermal growth factor receptor structural alterations in gastric cancer BMC Cancer 2008 8 10 10.1186/1471-2407-8-10 PMC2244615 18199332

[B24] Tsugawa K Yonemura Y Hirono Y Fushida S Kaji M Miwa K Miyazaki I Yamamoto H Amplification of the c-met, c-erbB-2 and epidermal growth factor receptor gene in human gastric cancers: correlation to clinical features Oncology 1998 55 5 475 481 10.1159/000011898 9732228

[B25] Han SW Oh DY Im SA Park SR Lee KW Song HS Lee NS Lee KH Choi IS Lee MH Phase II study and biomarker analysis of cetuximab combined with modified FOLFOX6 in advanced gastric cancer Br J Cancer 2009 100 2 298 304 10.1038/sj.bjc.6604861 PMC2634707 19127259

[B26] Chung KY Shia J Kemeny NE Shah M Schwartz GK Tse A Hamilton A Pan D Schrag D Schwartz L Cetuximab shows activity in colorectal cancer patients with tumors that do not express the epidermal growth factor receptor by immunohistochemistry J Clin Oncol 2005 23 9 1803 1810 10.1200/JCO.2005.08.037 15677699

[B27] Bardelli A Siena S Molecular mechanisms of resistance to cetuximab and panitumumab in colorectal cancer J Clin Oncol 2010 28 7 1254 1261 10.1200/JCO.2009.24.6116 20100961

[B28] Benavente S Huang S Armstrong EA Chi A Hsu KT Wheeler DL Harari PM Establishment and characterization of a model of acquired resistance to epidermal growth factor receptor targeting agents in human cancer cells Clin Cancer Res 2009 15 5 1585 1592 10.1158/1078-0432.CCR-08-2068 PMC2903727 19190133

[B29] Di Nicolantonio F Martini M Molinari F Sartore-Bianchi A Arena S Saletti P De Dosso S Mazzucchelli L Frattini M Siena S Wild-type BRAF is required for response to panitumumab or cetuximab in metastatic colorectal cancer J Clin Oncol 2008 26 35 5705 5712 10.1200/JCO.2008.18.0786 19001320

[B30] Qian X Karpova T Sheppard AM McNally J Lowy DR E-cadherin-mediated adhesion inhibits ligand-dependent activation of diverse receptor tyrosine kinases Embo J 2004 23 8 1739 1748 10.1038/sj.emboj.7600136 PMC394229 15057284

[B31] Witta SE Gemmill RM Hirsch FR Coldren CD Hedman K Ravdel L Helfrich B Dziadziuszko R Chan DC Sugita M Restoring E-cadherin expression increases sensitivity to epidermal growth factor receptor inhibitors in lung cancer cell lines Cancer Res 2006 66 2 944 950 10.1158/0008-5472.CAN-05-1988 16424029

[B32] Becker KF Atkinson MJ Reich U Becker I Nekarda H Siewert JR Hofler H E-cadherin gene mutations provide clues to diffuse type gastric carcinomas Cancer Res 1994 54 14 3845 3852 8033105

[B33] Gamboa-Dominguez A Dominguez-Fonseca C Chavarri-Guerra Y Vargas R Reyes-Gutierrez E Green D Quintanilla-Martinez L Luber B Busch R Becker KF E-cadherin expression in sporadic gastric cancer from Mexico: exon 8 and 9 deletions are infrequent events associated with poor survival Hum Pathol 2005 36 1 29 35 10.1016/j.humpath.2004.09.020 15712179

[B34] Bremm A Walch A Fuchs M Mages J Duyster J Keller G Hermannstadter C Becker KF Rauser S Langer R Enhanced activation of epidermal growth factor receptor caused by tumor-derived E-cadherin mutations Cancer Res 2008 68 3 707 714 10.1158/0008-5472.CAN-07-1588 18245470

[B35] Mateus AR Seruca R Machado JC Keller G Oliveira MJ Suriano G Luber B EGFR regulates RhoA-GTP dependent cell motility in E-cadherin mutant cells Hum Mol Genet 2007 16 13 1639 1647 10.1093/hmg/ddm113 17510211

[B36] Machado JC Oliveira C Carvalho R Soares P Berx G Caldas C Seruca R Carneiro F Sobrinho-Simoes M E-cadherin gene (CDH1) promoter methylation as the second hit in sporadic diffuse gastric carcinoma Oncogene 2001 20 12 1525 1528 10.1038/sj.onc.1204234 11313896

[B37] Ascano JJ Frierson H Jr Moskaluk CA Harper JC Roviello F Jackson CE El-Rifai W Vindigni C Tosi P Powell SM Inactivation of the E-cadherin gene in sporadic diffuse-type gastric cancer Mod Pathol 2001 14 10 942 949 10.1038/modpathol.3880416 11598162

[B38] Machado JC Soares P Carneiro F Rocha A Beck S Blin N Berx G Sobrinho-Simoes M E-cadherin gene mutations provide a genetic basis for the phenotypic divergence of mixed gastric carcinomas Lab Invest 1999 79 4 459 465 10211998

[B39] Uchikado Y Okumura H Ishigami S Setoyama T Matsumoto M Owaki T Kita Y Natsugoe S Increased Slug and decreased E-cadherin expression is related to poor prognosis in patients with gastric cancer Gastric Cancer 2011 14 1 41 49 10.1007/s10120-011-0004-x 21274735

[B40] Walch A Seidl S Hermannstadter C Rauser S Deplazes J Langer R von Weyhern CH Sarbia M Busch R Feith M Combined analysis of Rac1, IQGAP1, Tiam1 and E-cadherin expression in gastric cancer Mod Pathol 2008 21 5 544 552 10.1038/modpathol.2008.3 18246045

