# Supplementary material for: Biomarker analysis of cetuximab plus oxaliplatin/leucovorin/5-fluorouracil in first-line metastatic gastric and oesophago-gastric junction cancer: results from a phase II trial of the Arbeitsgemeinschaft Internistische Onkologie (AIO)
Source: BMC Cancer. 2011 Dec 7;11:509. doi: 10.1186/1471-2407-11-509 (PMC3252322; doi:10.1186/1471-2407-11-509)
Supplement: Additional file 1 — The file Supplementary-BMC Cancer in the PDF format contains supplementary results, methods and tables. [file 1471-2407-11-509-S1.PDF]

Biomarker analysis of cetuximab plus oxaliplatin/leucovorin/5-fluorouracil in first-line metastatic gastric and oesophago-gastric junction cancer: results from a phase II trial of the Arbeitsgemeinschaft Internistische Onkologie (AIO)

Birgit Luber, Joëlle Deplazes, Gisela Keller, Axel Walch, Sandra Rauser, Martin Eichmann, Rupert Langer, Heinz Höfler, Susanna Hegewisch-Becker, Gunnar Folprecht, Ewald Wöll, Thomas Decker, Esther Endlicher, Sylvie Lorenzen, Falko Fend, Christian Peschel, Florian Lordick

Supplementary Results

Relationship between *EGFR* gene copy numbers and therapy response

The characteristics of the 8 patients with increased *EGFR* copy number ($\geq$4.0) in their tumours are summarised in Supplementary Table S6. Interestingly, all 6 of 8 patients with primary tumours located in the OGJ responded to treatment, while two patients with cancer in other parts of the stomach were therapy resistant. This result suggested that patients with tumours located in the OGJ may have a more favourable outcome than patients with gastric cancer. To test this hypothesis, we assessed the clinical outcome of patients with OGJ tumours in terms of OS and TTP (Supplementary Table S7). We found that *EGFR* gene copy number ($\geq$4.0) was significantly associated with OS in Kaplan-Meier survival time analysis (log-rank *P*=0.035) and showed a trend toward an association in univariate Cox regression analysis (HR 0.2, 95% CI: 0-1.0; *P*=0.052) in this subgroup of patients. Additionally, it was possible to establish a correlation between increased *EGFR* gene copy number ($\geq$4.0) and extended TTP in Kaplan-Meier analysis (log-rank *P*=0.004) and in univariate Cox regression analysis (*P*=0.019) with only a 0.1-fold relative risk for

tumour progression in these patients (95% CI=0-0.7). Moreover, *EGFR* gene copy number ($\geq$4.0) in patients with tumours in the OGJ showed a trend toward an association with ORR (*P*=0.109) but not with CBR.

Immunohistochemical analysis of EGFR, pEGFR, pAkt, pMAPK and E-cadherin

Expression of EGFR was not detectable in 13 of 38 cases (34.2%). The staining intensity for EGFR was weak in 16 cases (42.1%, score 1+), moderate in 6 cases (15.8%, score 2+) and strong in 3 cases (7.9%, score 3+).

Absence of pEGFR was observed in 13 of 26 cases (50.0%). Most of the pEGFR-positive tumours (9 cases, 34.6%) showed weak staining intensity (score 1+), while moderate or strong staining was detected only in 3 cases (11.5%, score 2+) and 1 case (3.8%, score 3+), respectively. Heterogeneous staining of neoplastic cells was frequently observed for EGFR and pEGFR, with highly positive tumour cells adjacent to completely negative tumour cells.

Nuclear pAkt staining of neoplastic cells was observed for all 28 investigated tumours, and the reactivity was weak in 5 cases (17.9%, score 1+), moderate in 20 cases (71.4%, score 2+) and strong in 3 cases (10.7%, score 3+).

Complete absence of pMAPK reactivity was found in only one of 25 cases (4.0%). In most of the tumours (15 cases, 60.0%), the intensity of cytoplasmic or nuclear pMAPK staining was moderate (score 2+), whereas it was weak in 5 cases (20.0%, score 1+) and strong in 4 cases (16.0%, score 3+).

Membranous staining of E-cadherin was detectable in 24 of 25 evaluable patients (96.0%). Complete absence of E-cadherin reactivity was found in only one case (4.0%). In most of the tumours (14 cases, 56.0%), moderate E-cadherin staining at the cellular membrane was detected (score 2+). Several tumours were weakly- (4 cases (16.0%), score 1+) or strongly-positive (6 cases (24.0%), score 3+).

Mutations and polymorphisms of the *CDH1* gene

Several previously described *CDH1* polymorphisms were detected at nucleotide positions c531+10 in intron 4 (Patient 17), nucleotide position c1680 in exon 11 (Patient 6), nucleotide position c2253 in exon 14 (Patient 17), and nucleotide position c2292 in exon 14 (Patient 30) [1-4]. Another intronic *CDH1* polymorphism was found at nucleotide position c1009-4 (Patient 11) and this polymorphism was not described before. We found that known splice-sites were not affected by this intronic variation and immunostaining with mutation-specific antibodies that react with mutant E-cadherin proteins lacking exons 8 or 9 was negative.

Two *CDH1* missense mutations in exon 9 were described in the manuscript (Patients 11 and 27) [5]. The data bases www.sanger.ac.uk and www.ensembl.org were used.

Supplementary Methods

Fluorescence *in situ* hybridisation

The commercially available LSI EGFR SpectrumOrange/CEP 7 SpectrumGreen Dual Color Probe hybridises to the band region 7p12 in SpectrumOrange and the centromere of chromosome 7 (7p11.1-q11.1, D7Z1 locus) in SpectrumGreen. Between 50 and 100 whole (not cut) tumour cell nuclei per sample were analysed.

Immunohistochemical analysis

A manual staining protocol was used for pEGFR, pAkt and pMAPK and staining was performed using the following antibody dilutions: pEGFR 1:50, pAkt 1:250, pMAPK 1:100 and E-cadherin 1:500. Antigen retrieval was performed with a citrate buffer of pH 6 using a microwave (for pEGFR, 2 x 10 min) or a pressure cooker (for pMAPK, pAkt and E-cadherin, 7 min). A peroxidase block (3 % H_20_2 for 15 min at room temperature), an avidin biotin block (Vectastain, 2 x 15 min at room temperature) and blocking with 5 % anti-goat serum in Dako dilution solution (1 h, room temperature) were performed. Staining was carried out with LSAB-DAB from Dako Diagnostika GmbH (Hamburg, Germany).

As positive controls, we used formalin-fixed and paraffin-embedded cell pellets from EGF-treated A431 cells (for pEGFR) or MDA-MB-435S cells transfected with E-cadherin cDNA [6] (for pAkt, pMAPK and E-cadherin).

Reactivity score and interpretation of the immunohistochemical staining

For the analysis of pEGFR staining, tumours were considered negative when no staining or membrane staining in <10% neoplastic cells was observed. Weak

complete and/or incomplete membrane staining of pEGFR in >10% neoplastic cells was considered 1+ positive, moderate complete and/or incomplete membrane staining in >10% neoplastic cells was considered 2+ positive and strong complete and/or incomplete membrane staining in >10% neoplastic cells was considered 3+ positive.

For the analysis of pAkt staining, tumours were considered negative when no staining or nuclear staining in <10% neoplastic cells was detected. Weak pAkt staining in >10% neoplastic cells was considered 1+ positive, staining in >10% neoplastic cells was considered 2+ positive and strong staining in >10% neoplastic cell was considered 3+ positive.

For the evaluation of pMAPK levels, tumours were classified as negative when no staining or cytoplasmic or nuclear staining in <10% neoplastic cells was observed. Weak pMAPK staining in >10% neoplastic cells was classified as 1+ positive, staining in >10% neoplastic cells was classified as 2+ positive and strong staining in >10% neoplastic cells was classified as 3+ positive.

Finally, for the interpretation of E-cadherin staining, tumours were considered negative when no staining was detectable. Membranous E-cadherin staining in <10% neoplastic cells was considered 1+ positive, in between 10% and 50% of neoplastic cells was classified as 2+ positive and in >50% of neoplastic cells was considered 3+ positive.

DNA extraction

Genomic DNA was isolated from paraffin-embedded gastric and OGJ carcinoma tissue sections. Tumourous areas were marked on haematoxylin-eosin stained slides and manually dissected from 5-μm sections (tumour cell content >50%). Paraffin-embedded tissues were dewaxed with xylene, rehydrated through ethanol and resuspended in 200 μl 50 mM Tris-HCl pH 8.5, 1 mM EDTA, 0.5 % Tween 20 and 0.2 mg/ml proteinase K. After 3 h incubation at 55°C, proteinase K was inactivated by boiling for 10 min. Aliquots were used for mutation analysis of *CDH1* and *BRAF*.

***CDH1* mutation analysis**

CDH1 mutation analysis was carried out following PCR amplification of exons 2-16 with DNA isolated from tumour cells after manual dissection from formalin-fixed, paraffin-embedded tumour sections as described above. DHPLC is based on DNA heteroduplex formation between wild-type and mutant DNA and separation of heteroduplex from homoduplex molecular species by means of ion-pair reverse phase HPLC to detect mutations. DHPLC analysis was performed according to the method described by Oefner and Underhill [7] with an automated DHPLC analysis system (Transgenomic, Omaha, Nebraska).

The primers for direct sequencing of exon 9 of *CDH1* were previously described [8]. PCR products were purified from agarose gels using a gel extraction kit (Qiagen, Hilden, Germany). For cycle sequencing, the Ready Reaction Big Dye Terminator Cycle Sequencing kit (Applied Biosystems, Perkin-Elmer, Foster City, CA) and an automated sequencing system (3130 Genetic Analyzer, Applied Biosystems) were used. In total, 15 of 22 analysed tumours were of the non-intestinal type (diffuse or mixed type). The *CDH1* missense mutations occurred in 2 out of 15 (13%) of these

histological subtypes. The number of the National Center for Biotechnology Information (NCBI) reference sequence was NM_004360.3.

Statistical analysis

TTP was considered to be the period from the day of study assignment to the date of any progression or last contact, as reported earlier [9]. Patients who had not progressed at the time of the final analysis were censored at the date of their last tumour assessment. OS was calculated from the day of assignment to death, as previously described [9]. Patients alive at the final survival analysis were censored using the last contact date. Duration of treatment was calculated as the period from the first to the last day of treatment. Follow-up was considered to be the period from the day of study assignment to the last contact.

Supplementary References

1. Berx G, Cleton-Jansen AM, Nollet F, de Leeuw WJ, van de Vijver M, Cornelisse C, van Roy F: **E-cadherin is a tumour/invasion suppressor gene mutated in human lobular breast cancers**. *Embo J* 1995, **14**(24):6107-6115.

2. Berx G, Cleton-Jansen AM, Strumane K, de Leeuw WJ, Nollet F, van Roy F, Cornelisse C: **E-cadherin is inactivated in a majority of invasive human lobular breast cancers by truncation mutations throughout its extracellular domain**. *Oncogene* 1996, **13**(9):1919-1925.

3. Berx G, Becker KF, Hofler H, van Roy F: **Mutations of the human E-cadherin (CDH1) gene**. *Hum Mutat* 1998, **12**(4):226-237.

4. Risinger JI, Berchuck A, Kohler MF, Boyd J: **Mutations of the E-cadherin gene in human gynecologic cancers**. *Nat Genet* 1994, **7**(1):98-102.

5. Machado JC, Oliveira C, Carvalho R, Soares P, Berx G, Caldas C, Seruca R, Carneiro F, Sobrinho-Simoes M: **E-cadherin gene (CDH1) promoter methylation as the second hit in sporadic diffuse gastric carcinoma**. *Oncogene* 2001, **20**(12):1525-1528.

6. Handschuh G, Candidus S, Luber B, Reich U, Schott C, Oswald S, Becke H, Hutzler P, Birchmeier W, Hofler H *et al*: **Tumour-associated E-cadherin mutations alter cellular morphology, decrease cellular adhesion and increase cellular motility**. *Oncogene* 1999, **18**(30):4301-4312.

7. Oefner PJ, Underhill PA: **DNA mutation detection using denaturing high-performance liquid chromatography (DHPL)**. *In Dracopoli NC, Haines JL, Korff BR, Morton CC, Seidman CE, Seidman JG, Moir DT, Smith DR (eds): Current Protocols in Human Genetics New York, Wiley* 1998, **suppl 19**:7.10.11–17.10.12.

8. Becker KF, Reich U, Schott C, Becker I, Berx G, van Roy F, Höfler H: **Identification of eleven novel tumor-associated E-cadherin mutations. Mutations in brief no. 215. Online.** *Hum Mutat* 1999, **13**(2):171.

9. Lordick F, Luber B, Lorenzen S, Hegewisch-Becker S, Folprecht G, Woll E, Decker T, Endlicher E, Rothling N, Schuster T *et al*: **Cetuximab plus oxaliplatin/leucovorin/5-fluorouracil in first-line metastatic gastric cancer: a phase II study of the Arbeitsgemeinschaft Internistische Onkologie (AIO)**. *Br J Cancer* 2010, **102**(3):500-505.

Supplementary Tables

Table S1 Correlation analysis using the Pearson test

		**EGFR** IHC n=38	**pEGFR** IHC n=26	**pAkt** IHC n=28	**pMAPK** IHC n=25	**E-cad** IHC n=25	**EGFR** FISH n=35	**CEP7** FISH n=35	**EGFR/ CEP7** FISH n=35
**EGFR** IHC	**cc**	**-**	0.405*	-0.060	0.144	0.328	0.367*	0.313	0.157
	P		**0.040**	0.761	0.491	0.109	**0.030**	0.067	0.367
**pEGFR** IHC	**cc**	0.405*	-	-0.052	0.100	0.155	0.119	0.158	-0.010
	P	**0.040**		0.799	0.642	0.471	0.580	0.460	0.962
**pAkt** IHC	**cc**	-0.060	-0.052	-	-0.024	0.286	-0.235	-0.060	-0.423*
	P	0.761	0.799		0.909	0.165	0.259	0.774	**0.035**
**pMAPK** IHC	**cc**	0.144	0.100	-0.024	-	0.150	0.271	0.318	-0.015
	P	0.491	0.642	0.909		0.483	0.200	0.131	0.945
**E-cad** IHC	**cc**	0.328	0.155	0.286	0.150	-	0.320	0.342	-0.047
	P	0.109	0.471	0.165	0.483		0.128	0.102	0.826
**EGFR** FISH	**cc**	0.367*	0.119	-0.235	0.271	0.320	-	0.925**	0.409*
	P	**0.030**	0.580	0.259	0.200	0.128		**<0.001**	**0.013**
**CEP7** FISH	**cc**	0.313	0.158	-0.060	0.318	0.342	0.925**	-	0.052
	P	0.067	0.460	0.774	0.131	0.102	**<0.001**		0.763
**EGFR/ CEP7** FISH	**cc**	0.157	-0.010	-0.423*	-0.015	-0.047	0.409*	0.052	-
	P	0.367	0.962	**0.035**	0.945	0.826	**0.013**	0.763	

Abbreviations: E-cad: E-cadherin; cc: correlation coefficient; *P*: value significance (two-sided)
*The correlation is significant at the niveau 0.05 (two-sided).
** The correlation is significant at the niveau 0.01 (two-sided).

Table S2 Correlation analysis using the Spearman`s rho test

		**EGFR** IHC n=38	**pEGFR** IHC n=26	**pAkt** IHC n=28	**pMAPK** IHC n=25	**E-cad** IHC n=25	***EGFR*** FISH n=36	***CEP7*** FISH n=36	***EGFR/ CEP7*** FISH n=36
**EGFR** IHC	**cc**	**-**	0.369	-0.045	0.159	0.373	0.241	0.236	0.206
	P		0.064	0.820	0.448	0.066	0.163	0.173	0.234
**pEGFR** IHC	**cc**	0.369	-	-0.045	0.032	0.151	0.254	0.229	0.034
	P	0.064		0.828	0.881	0.481	0.232	0.282	0.874
**pAkt** IHC	**cc**	-0.045	-0.045	-	-0.016	0.237	-0.097	0.111	-0.403*
	P	0.820	0.828		0.940	0.254	0.646	0.596	**0.046**
**pMAPK** IHC	**cc**	0.159	0.032	-0.016	-	0.182	0.343	0.317	-0.018
	P	0.448	0.881	0.940		0.394	0.100	0.131	0.932
**E-cad** IHC	**cc**	0.373	0.151	0.237	0.182	-	0.385	0.334	-0.125
	P	0.066	0.481	0.254	0.394		0.063	0.111	0.559
EGFR FISH	**cc**	0.241	0.254	-0.097	0.343	0.385	-	0.868**	0.506**
	P	0.163	0.232	0.646	0.100	0.063		**<0.001**	**0.002**
CEP7 FISH	**cc**	0.236	0.229	0.111	0.317	0.334	0.868**	-	0.194
	P	0.173	0.282	0.596	0.131	0.111	**<0.001**		0.257
EGFR/ CEP7 FISH	**cc**	0.206	0.034	-0.403*	-0.018	-0.125	0.506**	0.194	-
	P	0.234	0.874	**0.046**	0.932	0.559	**0.002**	0.257	

Abbreviations: E-cad: E-cadherin; cc: correlation coefficient; *P*: value significance (two-sided)
*The correlation is significant at the niveau 0.05 (two-sided).
** The correlation is significant at the niveau 0.01 (two-sided).

Table S3 Correlations with overall survival (OS)

		Kaplan Meier			Cox regression (univariate)		
Variable	Score	Median	95% CI	LR *P*	HR	95% CI	*P*
**EGFR**<br>IHC<br>n=38	0<br>1, 2, 3	480<br>250	279-681<br>137-363	0.213	1.8	0.7-4.6	0.219
**pEGFR**<br>IHC<br>n=26	0<br>1, 2 ,3	490<br>285	258-722<br>162-408	0.619	1.3	0.4-3.8	0.620
**pAkt**<br>IHC<br>n=28	1<br>2, 3	-<br>480	-<br>275-685	0.920	1.1	0.2-4.8	0.920
**pMAPK**<br>IHC<br>n=25	0, 1<br>2, 3	106<br>480	-<br>176-784	0.485	0.6	0.2-2.4	0.489
**E-cadherin**<br>IHC<br>n=25	0, 1, 2<br>3	480<br>-	121-839<br>-	0.124	0.3	0.1-1.5	0.141
EGFR<br>FISH<br>n=36	<4.0<br>≥4.0	247<br>-	154-340<br>-	**0.011**	0.2	0.0-0.8	**0.022**
CEP7<br>FISH<br>n=36	<3.0<br>≥3.0	232<br>480	138-326<br>429-531	**0.025**	0.4	0.2-0.9	**0.030**
EGFR/CEP7<br>FISH<br>n=36	Diploid<br>(ratio 1.0)<br>Non-<br>diploid	250<br>456	204-296<br>214-698	0.137	0.5	0.2-1.3	0.144

Abbreviations: HR: hazard ratio; LR *P*: log-rank *P*
-: estimation was not possible

Table S4 Correlations with time to progression (TTP)

		Kaplan Meier			Cox regression (univariate)		
Variable	Score	Median	95% CI	LR *P*	HR	95% CI	*P*
**EGFR**<br>IHC<br>n=32	0<br>1, 2, 3	287<br>212	130-444<br>128-296	0.092	2.0	0.9-4.7	0.100
**pEGFR**<br>IHC<br>n=21	0<br>1, 2 ,3	291<br>121	245-337<br>6-236	**0.018**	4.0	1.2-13.9	**0.027**
**pAkt**<br>IHC<br>n=23	1<br>2, 3	112<br>258	0-291<br>208-308	0.192	0.5	0.1-1.5	0.203
**pMAPK**<br>IHC<br>n=20	0, 1<br>2, 3	260<br>258	0-536<br>195-321	0.775	0.8	0.2-3.1	0.775
**E-cadherin**<br>IHC<br>n=21	0, 1, 2<br>3	186<br>265	0-454<br>234-296	0.990	1.0	0.3-3.2	0.990
EGFR<br>FISH<br>n=29	<4.0<br>≥4.0	186<br>291	110-262<br>224-358	0.216	0.5	0.2-1.5	0.225
CEP7<br>FISH<br>n=29	<3.0<br>≥3.0	164<br>265	103-225<br>210-320	0.158	0.6	0.2-1.3	0.165
EGFR/CEP7<br>FISH<br>n=29	Diploid<br>(ratio 1.0)<br>Non-<br>diploid	142<br>260	84-200<br>187-333	**0.045**	0.4	0.1-1.0	0.056

Abbreviations: HR: hazard ratio; LR *P*: log-rank *P*

Table S5 Correlations with ORR and CBR

Variable	Score	No. of patients (%)	ORR *P* value	CBR *P* value
**EGFR**<br>IHC<br>n=36	0<br>1, 2, 3	13 (36%)<br>23 (64%)	0.501	0.686
**pEGFR**<br>IHC<br>n=25	0<br>1, 2 ,3	12 (48%)<br>13 (52%)	**0.004**	0.160
**pAkt**<br>IHC<br>n=27	1<br>2, 3	4 (15%)<br>23 (85%)	>0.999	>0.999
**pMAPK**<br>IHC<br>n=24	0, 1<br>2, 3	6 (25%)<br>18 (75%)	0.665	0.618
**E-cadherin**<br>IHC<br>n=24	0, 1, 2<br>3	18 (75%)<br>6 (25%)	0.665	0.629
EGFR<br>FISH<br>n=34	<4.0<br>≥4.0	26 (76%)<br>8 (24%)	0.444	>0.999
CEP7<br>FISH<br>n=34	<3.0<br>≥3.0	18 (53%)<br>16 (47%)	0.172	0.660
EGFR/CEP7<br>FISH<br>n=34	Diploid<br>(ratio 1.0)<br>Non-diploid	7 (21%)<br><br>27 (79%)	>0.999	0.306

Abbreviations: ORR: overall response rate; CBR: clinical benefit rate
Fisher´s exact test, two-sided

Table S6 Cases with *EGFR* gene copy number ≥4.0

			FISH analysis			
Patient No	Primary tumour localisation	Histologic classification (Laurén)	*EGFR*	*CEP7*	*EGFR/ CEP7* ratio	Best response
1	OGJ	Non-intestinal	4.14	3.86	1.07	CR
2	S	Intestinal	4.22	4.08	1.03	SD
7	OGJ	Intestinal	4.70	3.82	1.23	PR
8	OGJ	Intestinal	4.16	3.7	1.12	PR
9	OGJ	Intestinal	8.2	6.04	1.36	PR
18	OGJ	Intestinal	5.78	5.18	1.12	PR
23	S	Non-intestinal	4.0	3.84	1.04	PD
30	OGJ	Non-intestinal	4.2	3.98	1.06	CR

Abbreviations: CR: complete response; OGJ: Oesophago-gastric junction; NE: not evaluable; PD: progressive disease; PR: partial response; SD: stable disease; S: stomach

Table S7 Correlations with OS and TTP in patients with OGJ cancer

		Kaplan Meier			Cox regression (univariate)		
Variable	Score	Median	95% CI	LR *P*	HR	95% CI	*P*
		OS					
EGFR FISH n=16	<4.0<br>≥4.0	247<br>-	175-319<br>-	**0.035**	0.2	0-1.0	0.052
		TTP					
EGFR FISH n=16	<4.0<br>≥4.0	189<br>320	94-284<br>275-365	**0.004**	0.1	0-0.7	**0.019**

Abbreviations: HR: hazard ratio; LR *P*: log-rank *P*
-: estimation was not possible

Table S8 Frequency of immunohistochemical staining

	Score			
Protein	0<br>n (%)	1<br>n (%)	2<br>n (%)	3<br>n (%)
**EGFR**<br>Total: n=38	13 (34.2%)	16 (42.1%)	6 (15.8%)	3 (7.9%)
**pEGFR**<br>Total: n=26	13 (50.0%)	9 (34.6%)	3 (11.5%)	1 (3.8%)
**pAkt**<br>Total: n=28	-	5 (17.9%)	20 (71.4%)	3 (10.7%)
**pMAPK**<br>Total n=25	1 (4.0%)	5 (20.0%)	15 (60.0%)	4 (16.0%)
**E-cadherin**<br>Total n=25	1 (4.0%)	4 (16.0%)	14 (56.0%)	6 (24.0%)

Table S9 Mutations and polymorphisms of the *CDH1* gene

	Patient No.	Nucleotide position	Nucleotide exchange	Sequence variation	Codon No.	Amino acid exchange	Reference
Intron 4	17	c.531+10	G>C	SNP	-	-	Berx et al., 1995,1996,1998
Intron 7	11	c.1009-4	G>A	Intronic variation	-	-	n.d.
Exon 9	27	c.1204	G>C	Missense mutation	402	D>H	Nucleotide exchange: n.d.<br><br>Codon exchange: Machado et al., 2001
	11	c.1223	C>T	Missense mutation	408	A>V	n.d.
Exon 11	6	c.1680	G>C	SNP	560	-	Risinger et al., 1994<br><br>Berx et al., 1995,1996,1998
Exon 14	17	c.2253	C>T	SNP	751	-	Risinger et al., 1994<br><br>Berx et al., 1995,1996,1998
	30	c.2292	C>T	SNP	764	-	Risinger et al., 1994

Abbreviations: SNP: single nucleotide polymorphism; n.d.: not described

Table S10 Characteristics and response of patients with genomic mutations

Patient No.	Primary tumor localisation	Histologic classification (Laurén)	Mutations of		Best response
			CDH1	*KRAS*	
36	OGJ	Intestinal	-	+	SD
11	Stomach	Non-intestinal	+	-	NE
27	Stomach	Non-intestinal	+	-	PD

Abbreviations: OGJ: Oesophago-gastric junction; NE: not evaluable; PD: progressive disease; SD: stable disease
